# Closing the sea surface mixed layer temperature budget from in situ observations alone: Operation Advection during BoBBLE

**DOI:** 10.1038/s41598-020-63320-0

**Published:** 2020-04-27

**Authors:** V. Vijith, P. N. Vinayachandran, Benjamin G. M. Webber, Adrian J. Matthews, Jenson V. George, Vijay Kumar Kannaujia, Aneesh A. Lotliker, P. Amol

**Affiliations:** 1 Centre for Atmospheric and Oceanic Sciences, Indian Institute of Science, Bangalore, India; 2 Present Address: School of Marine Sciences, Cochin University of Science and Technology, Kochi, India; 3 Climatic Research Unit, School of Environmental Sciences, Centre for Ocean and Atmospheric Sciences, University of East Anglia, Norwich, UK; 4 School of Environmental Sciences and School of Mathematics, Centre for Ocean and Atmospheric Sciences, University of East Anglia, Norwich, UK; 5 CSIR-National Institute of Oceanography, Goa, India; 6 Indian National Centre for Ocean Information Services, Hyderabad, India; 7 CSIR-National Institute of Oceanography, Regional Center, Visakhapatnam, India

**Keywords:** Physical oceanography, Fluid dynamics

## Abstract

Sea surface temperature (SST) is a fundamental driver of tropical weather systems such as monsoon rainfall and tropical cyclones. However, understanding of the factors that control SST variability is lacking, especially during the monsoons when *in situ* observations are sparse. Here we use a ground-breaking observational approach to determine the controls on the SST variability in the southern Bay of Bengal. We achieve this through the first full closure of the ocean mixed layer energy budget derived entirely from *in situ* observations during the Bay of Bengal Boundary Layer Experiment (BoBBLE). Locally measured horizontal advection and entrainment contribute more significantly than expected to SST evolution and thus oceanic variability during the observation period. These processes are poorly resolved by state-of-the-art climate models, which may contribute to poor representation of monsoon rainfall variability. The novel techniques presented here provide a blueprint for future observational experiments to quantify the mixed layer heat budget on longer time scales and to evaluate these processes in models.

## Introduction

The calculation of the heat budget for the ocean surface mixed layer (ML) is an important task since it determines sea surface temperature (SST). Variability in tropical SST plays an important role in determining atmospheric convection over the tropical ocean^1,2^ and in turn influences large-scale ocean-atmosphere interaction processes such as the Asian monsoon^3^, El Niño^4^, tropical cyclones^5,6^, expansion of sea ice in the Antarctic^7^ and even pantropical climate interactions^8^. Therefore, calculation and diagnosis of the ML heat budget will reveal key processes that influence such coupled phenomena. However, no full closure of the ocean heat budget exists using *in situ* measurements alone, owing to the operational and scientific challenges involved, particularly with regard to measuring horizontal gradients of temperature.

SST is determined by physical processes occurring at the sea surface and in the surface ML. Processes that drive the rate of change (tendency) of SST, often referred to as the terms of the heat-budget equation, are horizontal advection, horizontal and vertical mixing, entrainment and net surface heat flux. Mathematically these processes are represented as follows (See Methods).

1 $$\begin{array}{rcl}\mathop{\underbrace{\frac{\partial {T}_{a}}{\partial t}}}\limits_{\text{Tendency}} & = & \mathop{\underbrace{-\left({u}_{a}\frac{\partial {T}_{a}}{\partial x}+{v}_{a}\frac{\partial {T}_{a}}{\partial y}\right)}}\limits_{\text{Horizontal}\,\text{advection}}+\mathop{\underbrace{{\kappa }_{H}\left(\frac{{\partial }^{2}{T}_{a}}{\partial {x}^{2}}+\frac{{\partial }^{2}{T}_{a}}{\partial {y}^{2}}\right)}}\limits_{\text{Horizontal}\,\text{mixing}}-\mathop{\underbrace{\frac{1}{h}{\left[{\kappa }_{Z}\frac{\partial T}{\partial z}\right]}_{-h}}}\limits_{\text{Vertical}\,\text{mixing}}\\  &  & -\,\mathop{\underbrace{\left(\frac{{T}_{a}-{T}_{-h}}{h}\right)\left(\mathop{\underbrace{\frac{\partial h}{\partial t}}}\limits_{\text{ML}\,\text{tendency}}+\mathop{\underbrace{{u}_{-h}\frac{\partial h}{\partial x}+{v}_{-h}\frac{\partial h}{\partial y}}}\limits_{\text{Lateral}\,\text{induction}}+\mathop{\underbrace{{w}_{-h}}}\limits_{\text{Vertical}\,\text{advection}}\right)}}\limits_{\text{Entrainment}}+\mathop{\underbrace{\frac{{q}_{0}-{q}_{pen}}{{\rho }_{0}{c}_{p}h}}}\limits_{\text{Net}\,\text{heat}\,\text{flux}}\mathrm{}.\end{array}$$

Here T, ${\rho }_{0}$, $h$ and ${c}_{p}$ are the temperature, mean density, the mixed layer depth (MLD) and specific heat capacity of sea water, respectively. $u$ and $v$ denote the horizontal components of velocity. The suffix $a$ denotes a vertically-averaged (over the ML) quantity and the subscript −*h* denotes the quantity at the base of the ML (see Methods). ${q}_{0}$ is the net heat flux at the oceanic surface and ${q}_{pen}$ represents the penetrative loss of shortwave radiation. ${\kappa }_{H}$ and ${\kappa }_{Z}$ are the horizontal and vertical eddy diffusivities, respectively (see Methods).

To estimate this heat budget, *in situ* time-series observations at a single (ship or mooring) location are typically combined with satellite or model data sets, in order to obtain horizontal gradients^9–12^, but this approach introduces large uncertainties and cannot resolve important small-scale or short-term processes. Recent efforts^9–11^ used data from moored buoys^13–15^ but required air-sea fluxes, currents and temperature gradients from other data sources such as satellites and so could not resolve subsurface mixing, entrainment or other small scale ML processes. Additional uncertainty arises in the estimation of penetrative short wave radiation, when empirical formulas based on satellite-derived chlorophyll concentration are used^10,16^. The heat budget is often estimated over a constant depth, thus ignoring entrainment^12,17^. Further, estimates of the individual heat budget terms are usually not calculated simultaneously^10^, and a full closure of the equation is not usually feasible.

## Results

### Bay of Bengal Boundary Layer Experiment

**Figure 1:**
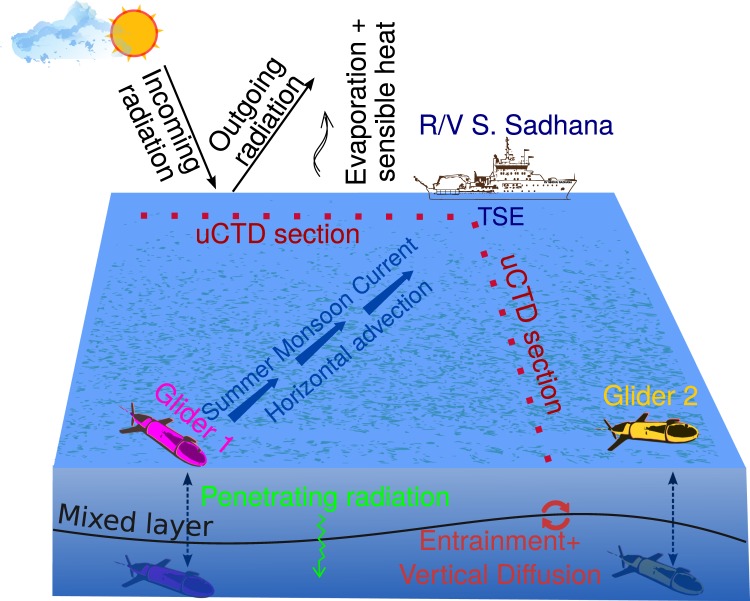
Schematic diagram illustrating the time-series measurements during the BoBBLE experiment and the mixed-layer processes in Eq. 1.

**Figure 2:**
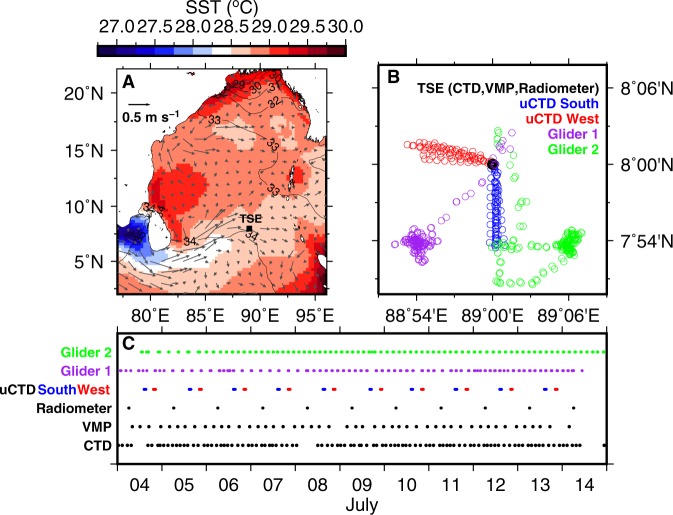
(**A**) Climatological (1993–2015) SST from Tropical Rainfall Measuring Mission (TRMM) Microwave Imager (TMI) and currents from Ocean Surface Current Analysis Real Time (OSCAR); the black square indicates the time-series location (TSE). (**B**) Locations of the measurement platforms (see legend). (**C**) Time (UTC) of measurement for each platform.

Here we use a groundbreaking approach (summarised schematically in Fig. 1) that combines high-resolution observations from ship-based and autonomous platforms to nearly close the surface ML heat budget and reveal the crucial importance of subsurface ocean processes for the evolution of SST in the Bay of Bengal (BoB), a region that has a prominent role in driving the Asian Summer Monsoon (ASM)^18^. Time series of oceanographic properties, including temperature, salinity, velocity, underwater radiation and subsurface mixing, along with surface fluxes of heat, were calculated from shipboard measurements onboard the *RV Sindhu Sadhana* in the southern BoB during the boreal summer monsoon of 2016 as part of the Bay of Bengal Boundary Layer Experiment (BoBBLE)^18^. Time-series measurements were made continuously for 11 days during 4–14 July at 89°E, 8°N (hereafter referred to as TSE; shown in Fig. 2A) using a CTD, uCTD, two ocean gliders, an Acoustic Doppler Current Profiler (ADCP), a Vertical Microstructure Profiler (VMP), an Automated Weather Station (AWS) and an underwater radiometer (see Methods). Figure 2B,C show, respectively, the locations and time of measurements of each instrument.

The horizontal advection term is a key process that is difficult to measure. To calculate this term, we adopted an Operation Advection (OA) strategy during BoBBLE, as follows. uCTD profiles were taken at intervals of approximately 1 km along westward and southward sections from TSE once per day, after dusk (Fig. 2B,C). Additionally, two ocean gliders were deployed in an ‘L’ shaped configuration, approximately 15 km long, with the gliders at the ends of the ‘L’ and the ship at the corner. The ‘L’ was oriented so that the first glider was positioned upstream of the ship in the direction of the climatological (northeastward) current, with the second glider located perpendicular to this direction (Figs. 1 and 2B). Temperature profiles were obtained approximately every 2 hours from the ship CTD and every 3 hours from the gliders throughout the 11-day occupation of TSE (Fig. 2C), allowing continuous and relatively high-frequency estimates of the horizontal temperature gradients. These were combined with current velocities from the shipboard ADCP to calculate the advection term. Independent estimates of the horizontal advection terms were obtained from the uCTD and gliders. Previous attempts with a uCTD or a CTD have involved surveying in a butterfly-shaped pattern^12,17^ and took about 1.5 days to complete, during which time the ocean state would have changed significantly. The OA strategy employed during the Continental Tropical Convergence Zone (CTCZ) Programme in the BoB^19^, measured CTD profiles at four edges of a ‘plus’ with the time-series location as the center but lacked simultaneity and completeness in measuring all required parameters. The OA strategy that was adopted during BoBBLE took only one hour to complete each uCTD leg. This ensured independent simultaneous measurements of the horizontal temperature gradients and advection term once per day.

**Figure 3:**
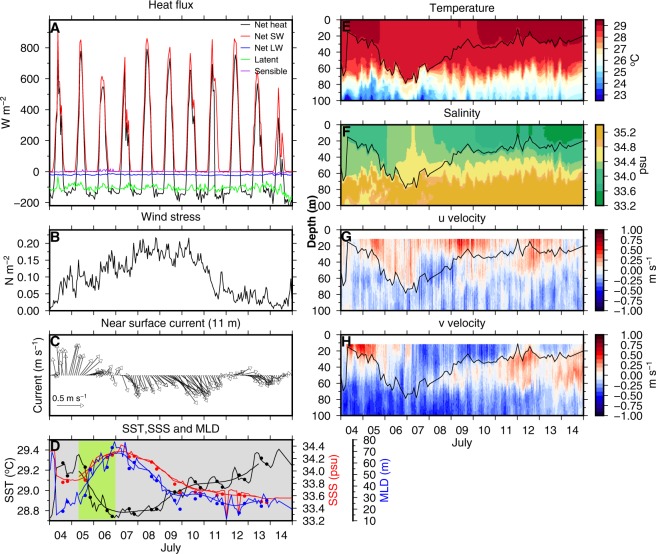
Time series of (**A**) surface heat fluxes, (**B**) wind stress, (**C**) near-surface current velocity, (**D**) SST, SSS and MLD. Vertical structure of (**E**) temperature, (**F**) salinity, (**G**) u and (**H**) v components of current velocity at TSE during 4–14 July 2016. 24 hours low-pass filtered SST, SSS and MLD are shown using thick curves in (**D**). Filled circles in (**D**) are from the daily measurements from the uCTD. Warming (cooling) events are highlighted using a grey (green) background colour in (**D**). MLD is shown using a black curve in (**E**–**H**). Time axis is in UTC.

The 11-day time-series observations were carried out during a period of suppressed atmospheric convection with high surface solar radiation and the surface heat flux balance was mainly between the solar radiation and latent heat flux (Fig. 3A). Wind stress was moderate to weak (<0.2 Nm^−2^) with the maximum observed on 8 July (Fig. 3B). During July, the strong northeastward Southwest Monsoon Current^18,20^ has been observed to advect cooler and saltier water to the TSE location (Fig. 3C,D)^21^. The near-surface currents were moderate (~0.35 m s^−1^) and flowed northeastward during the first three days of the observation and then reversed on 7 July (Fig. 3C). The zonal and meridional components of the currents were nearly uniform within the ML (Fig. 3G,H).

From 5–6 July, the SST cooled by 0.5 °C, from 29.2 °C to 28.8 °C (Fig. 3D,E), while the MLD deepened from 20–30 m to 60–70 m, eroding the sub-surface maxima in salinity and increasing the sea surface salinity (SSS) from $33.8$ to $34.2$ psu (Fig. 3D,F ^21^). This cooling and ML deepening phase did not coincide with the occurrence of a maximum in wind stress or with a net heat loss at the surface^21^. Hence, it cannot be explained directly by local surface forcing. Moreover, the cooling event occurred concurrently with the northeastward flow of near-surface currents. Hence, advection is likely to play a major role in this event. Following the cooling event, as the currents reversed and flowed southeastward, the SSS decreased, the ML shallowed and the sea surface warmed (Fig. 3C–F). The warming was superposed with a diurnal oscillation with an amplitude of ~0.1 °C^22^. By the end of the observations, the temperature, salinity and MLD were restored to their values at the beginning of the observations.

### Closure of the heat budget

**Figure 4:**
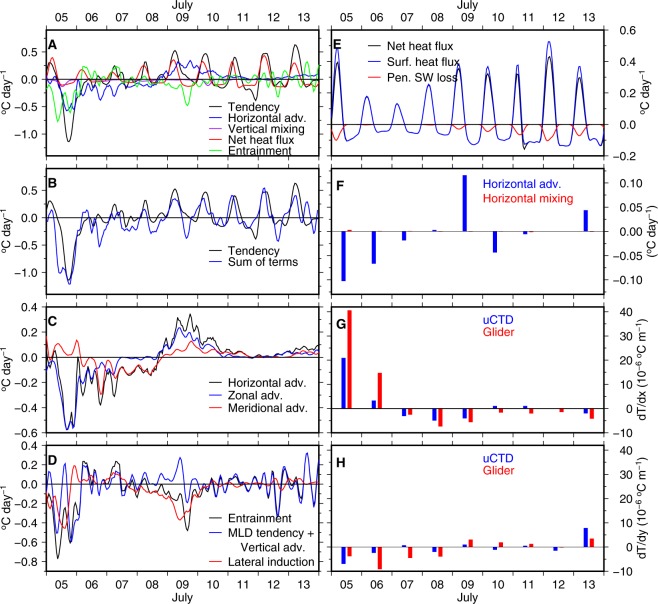
(**A**) Time series of the terms of the heat budget equation (Eq. 1). (**B**) Tendency and sum of the terms on the right hand side of Eq. 1. (**C**) Zonal and meridional components of the horizontal advection. (**D**) Components of the entrainment term (tendency of the MLD, lateral induction and vertical advection). (**E**) Components of the net heat flux term (surface heat flux and penetrating shortwave heat loss). The curves in (**A**–**E**) are smoothed using a 4-hour moving window to remove high frequency noises in the estimates. (**F**) Comparison between horizontal advection and horizontal mixing terms estimated using uCTD sections. (**G**) Comparison of the zonal gradient estimated using uCTD with gliders. (**H**) Same as (**G**), but for the meridional gradient.

The sum of ML-averaged (see Methods) horizontal advection, vertical mixing, net surface heat flux (corrected for the loss due to penetrating short wave radiation) and entrainment reproduces the overall pattern of the tendency term (Fig. 4A,B) with a correlation of $0.77$ and root mean square difference of 0.22 ± 0.4 °C day^−1^ (within 95% confidence interval). This is a remarkably good closure of the ML heat budget, especially given the magnitude of small-scale variability, and is unprecedented for such short time scales.

Our analysis reveals that oceanic processes such as horizontal advection and entrainment are important in determining the net tendency of the temperature, as was evident on 5 and 9 July. On 5 July, when the negative tendency reached its peak (~−1 °C day^−1^), the horizontal advection (~−0.5 °C day^−1^) and entrainment (~−0.5 °C day^−1^) were the predominant contributors (Fig. 4A). The decomposition of these two processes reveals that the advection was in the zonal direction (Fig. 4C), towards the east, and the entrainment was caused mainly by the lateral induction (Fig. 4D) that arises from the horizontal advection across a sloping ML base. On 9 July, the horizontal advection was strongly positive, but was opposed by strong negative entrainment (largely due to lateral induction), resulting in a weak temperature tendency (Fig. 4A).

Net heat flux contributed to the warming that occurred during 8–9 July, as was expected from previous studies^23^. The diurnal variation that was observed in the tendency during the warming period (7–13 July) was driven by the diurnal variation in the net heat flux (Fig. 4A,E). As the ML shallowed, the penetrating short wave loss at the base of the ML increased. On 12 July, when the ML was shallower than 30 m, the loss at the base of the ML was about $\mathrm{20 \% }$ of the radiation received at the sea surface (Fig. 4E). During these days, the chlorophyll fluorescence was low, indicating that the water was clear and allowed penetration of short-wave radiation below the ML (Fig. S1). The vertical (Fig. 4A) and horizontal mixing (Fig. 4F) were very weak throughout the observation period.

**Figure 5:**
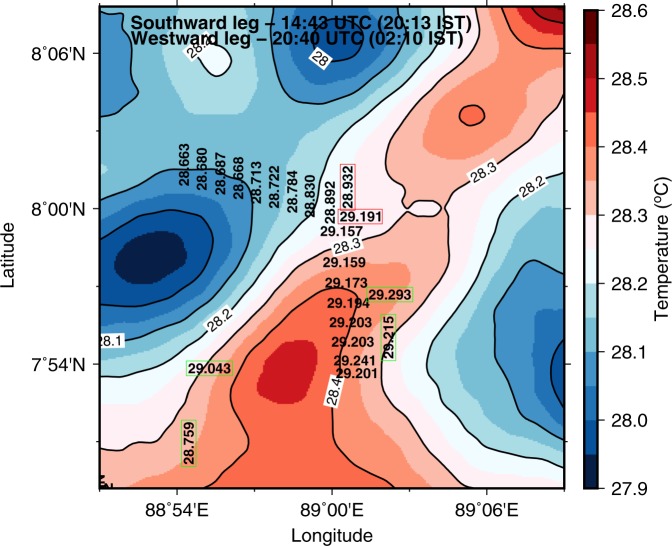
Distribution of ML-averaged temperature on 5 July measured by uCTD, CTD and gliders. Measurements from CTD and gliders are interpolated to the time of measurement of the uCTD profiles. Measurements from the CTD (gliders) are given in red (green) rectangular boxes. Values written in horizontal (vertical) direction corresponds to the southward (westward) section of uCTD. Colors are high resolution SST (approximately 1.1 km) from the Group of High Resolution Sea Surface Temperature (GHRSST) product. A bias of about 0.5 °C is seen between the satellite-derived SST and the *in situ* data, probably owing to aliasing of the high-frequency variability (diurnal cycle and trend) and cool-skin effect of measuring SST.

The temperature gradient was estimated using two different platforms, uCTD and gliders (Fig. 4G,H). Glider based estimates for $\partial T/\partial x$ were consistently larger in magnitude, for both positive and negative values of $\partial T/\partial x$. Whereas, for $\partial T/\partial y$, there was no consistent bias. These differences represent real small-scale variability in spatial gradients. A map of high-resolution satellite SST, superimposed with the individual temperature measurements from CTD, uCTD and gliders (Fig. 5), shows that there is large small-scale spatial variability in SST gradients even at a scale of 20 km that explains the different measurements from these configurations. These differences underline the value of having two complementary observations of spatial gradients and the importance of high spatial resolution for these measurements. Besides, *in situ* measurements by uCTD and gliders were conducted only to the west and south of TSE (Fig. 1B) and can induce biases in the estimates of temperature gradients. Such large small-scale variability in space and time and biases can result in residuals (Fig. 4B) while attempting the closure of the temperature equation.

## Discussion

In summary, we have demonstrated that a novel combination of ship-based and autonomous platforms can effectively close the surface ML heat budget at unprecedented temporal resolution from *in situ* measurements alone. The time-series observations were carried out during a convectively suppressed phase of the boreal summer intraseasonal oscillation^18^, a cloud-free period, during which the sea surface is likely to warm due to intense solar radiation. However, our estimates revealed that SST in the BoB is significantly modulated by lateral advection and entrainment. During the initial stage of the observation, these oceanic processes inhibited the surface warming and thus delayed the transition towards a convectively active phase over the central BoB. Hence, within the BoB, the oceanic processes are key mechanisms that set the basin-scale SST distribution and influence convection over the region^24,25^, and our results underline that these processes cannot be neglected at any scale. Notably, the synoptic-scale convective systems that originate in the BoB during the active phase of the ASM propagate westward or northwestward and cause rainfall over the land^3,26^.

However, such small scale processes are difficult to resolve in coupled climate models and may thus contribute to biases in simulation of the monsoon^27^. Forecasting monsoon rainfall at intraseasonal to seasonal time scales requires accurate simulation of SST evolution^28^, and inadequate representation of these processes may also hinder simulations of the influence of climate change on monsoon rainfall^27^. The proof-of-concept study presented here (summarised in Fig. 1) serves as a blueprint for future observational campaigns that aim at determining ML energy budget over longer time scales. Such studies will help to identify sources of model biases in simulating SST and its variability, and thus aid improvements in model simulations of coupled ocean-atmosphere processes. This approach may be used for studying monsoon processes as here, and also for other phenomena such as: the ocean component of the Madden-Julian Oscillation^29^ and interannual modes of variability such as El Niño-Southern Oscillation^30^ and the Indian Ocean Dipole^31^.

## Methods

### Instruments

Temperature and salinity in the upper 500 m of the water column at the time-series location, TSE (8°N, 89°E), were repeatedly measured at approximately 3 hourly intervals using a CTD (SeaBird Electronics, SBE 9/11+) deployed from RV Sindhu Sadhana. We deployed two gliders, equipped with CT sensors, about $16$ km southwest (7.9°N, 88.9°E) and southeast of TSE (7.9°N, 89.1°E), respectively. These gliders measured profiles from the surface to about 1000 m at a vertical resolution of 0.5–1 m. Temperature and salinity data from the gliders and CTD were optimally interpolated to a two-dimensional time-depth grid, with grid spacings of 1 hr and $1$ m. Further details are in Matthews *et al*.^22^. However, the effective temporal resolution of these gridded data sets is approximately 2–3 hr, corresponding to the approximate time intervals between glider and ship successive CTD profiles. Two vertical sections of temperature and salinity were measured, every day at around 1400 hours (UTC) and 1900 hours (UTC), respectively running southwards and westward of TSE, using a uCTD (Ocean Sciences-Teledyne underway CTD) towed by the ship. Each of these vertical sections was approximately $10$ km long and took approximately one hour to complete. We measured temperature and salinity profiles using the uCTD while the ship was sailing at a steady speed of 6 knots. The uCTD probe was allowed to drop freely for 2 minutes at a vertical rate of 1.5–2.5 m s^−1^ from the surface to a depth of 180–300 m, which encompassed the ML. Ocean current velocity was measured using an ADCP with an operational frequency of 150 kHz. The data were processed^32^ to derive current velocity in the top 11–180 m depth at a vertical resolution of 2 m and temporal resolution of 2 minutes. Data were corrected for mis-alignment angle and data collected during sudden accelerations were discarded. The data were then temporally averaged over a one-hour interval to be consistent with the CTD and glider data. To estimate vertical eddy diffusivity (*K*_*Z*_) of temperature, a loosely tethered VMP (Rockland Scientific, VMP-250), equipped with two airfoil shear probes and a CT sensor, was used. Vertical profiles were measured to a maximum of 250 m, five times a day (0330, 0730, 1200, 1800 and 2330 hours UTC), using the VMP. An AWS, onboard the vessel, installed at an approximate height of 15 m above the sea surface, gave measurements of wind velocity (RM Young), atmospheric temperature (YSI), pressure (Honeywell), relative humidity (Rotronic), and all the components of the surface heat fluxes (LI-COR infra-red gas analyzer with 3D sonic anemometer-based eddy covariance system). To quantify the penetrative shortwave radiation, a hyperspectral underwater radiometer (Satlantic HyperProII) was used. The instrument assembly was equipped with sensors measuring downwelling irradiance (Ed), upwelling radiance (Lu), chlorophyll fluorescence, coloured dissolved organic matter fluorescence, back-scattering, conductivity and temperature. Three profiles were measured using the radiometer in the upper 150 m every day between 0600 and 0700 hours (UTC) at TSE.

### Heat budget equation

The heat budget equation averaged in the space-time dependent oceanic ML, applying incommpressibility and Boussinesq approximations, is written as follows (See the Appendix of Moisan and Niiler, 1998^33^ for detailed derivation).

2 $$\begin{array}{l}\mathop{\underbrace{\frac{\partial {T}_{a}}{\partial t}}}\limits_{\text{Tendency}}+\mathop{\underbrace{\left({u}_{a}\frac{\partial {T}_{a}}{\partial x}+{v}_{a}\frac{\partial {T}_{a}}{\partial y}\right)}}\limits_{\text{Horizontal}\,\text{advection}}-\mathop{\underbrace{\frac{{\kappa }_{H}}{h}{\int }_{-h}^{0}\left(\frac{{\partial }^{2}T}{\partial {x}^{2}}+\frac{{\partial }^{2}T}{\partial {y}^{2}}\right)dz}}\limits_{\text{Horizontal}\,\text{turbulent}\,\text{mixing}}\\ \,+\,\mathop{\underbrace{\left(\frac{{T}_{a}-{T}_{-h}}{h}\right)\left(\frac{\partial h}{\partial t}+{u}_{-h}\frac{\partial h}{\partial x}+{v}_{-h}\frac{\partial h}{\partial y}+{w}_{-h}\right)}}\limits_{\text{Entrainment}}=\mathop{\underbrace{\frac{{q}_{0}-{q}_{-h}}{{\rho }_{0}{c}_{p}h}}}\limits_{\text{Net}\,\text{heat}\,\text{flux}}\mathrm{}.\end{array}$$

Here ${\rho }_{0}$, ${c}_{p}$, and $T$ are the density (assumed to be a constant, 1025 kg m^−3^), specific heat capacity (3940 J kg^−1^ °C^−1^), and temperature of seawater, respectively, and, $h$ is the MLD. The horizontal components of velocity are denoted by $u$ and $v$. Suffix $a$ denotes a vertically-averaged quantity in the ML and suffix −*h* denotes the quantity at the base of the ML. The vertical average of temperature and the horizontal components of velocity are defined as follows,

3 $${T}_{a}=\frac{1}{h}\,{\int }_{-h}^{0}\,T\,dz,\,{u}_{a}=\frac{1}{h}\,{\int }_{-h}^{0}\,u\,dz,\,{v}_{a}=\frac{1}{h}\,{\int }_{-h}^{0}\,v\,dz.$$

The terms in the Eq. 2, from left to right, are tendency (rate of change of temperature averaged over the MLD), horizontal advection, horizontal turbulent mixing, entrainment and net heat flux. The net heat flux comprises ${q}_{0}$, the net heat flux at the ocean surface, and ${q}_{-h}$, which represents the sum of penetrative loss of shortwave radiation (${q}_{pen}$) and vertical turbulent mixing at the base of the ML. Moisan and Niiler, 1998^33^, had integrated the temperature equation from a constant isotherm located below the thermocline to the surface and hence, ignored the penetrative shortwave radiation and entrainment. However, these terms are not negligible, if the lower limit of integration is the base of the ML. We adopt the most widely used parameterization based on the second derivative of temperature for the vertical and horizontal turbulent mixing. The vertical turbulent mixing term, averaged in the ML, is given by,

4 $$\frac{1}{h}\,{\int }_{-h}^{0}\,\frac{\partial }{\partial z}\left({\kappa }_{Z}\frac{\partial T}{\partial z}\right)dz=-\,\frac{1}{h}{\left[{\kappa }_{Z}\frac{\partial T}{\partial z}\right]}_{-h}$$

Here, ${\kappa }_{Z}$ is the vertical eddy diffusivity, estimated from the vertical microstructure measurements.

We estimate the second derivative of the temperature using the uCTD data. Assuming a spatially and temporally invariant horizontal eddy diffusivity (${\kappa }_{H}$), the horizontal turbulent mixing term, averaged in the ML is given by,

5 $$\frac{{\kappa }_{H}}{h}\,{\int }_{-h}^{0}\,\left(\frac{{\partial }^{2}T}{\partial {x}^{2}}+\frac{{\partial }^{2}T}{\partial {y}^{2}}\right)dz$$

Applying the Leibniz rule,

6 $${\int }_{-h}^{0}\,\frac{{\partial }^{2}T}{\partial {x}^{2}}dz=h\frac{{\partial }^{2}{T}_{a}}{\partial {x}^{2}}+({T}_{a}-{T}_{-h})\frac{{\partial }^{2}h}{\partial {x}^{2}}+2\frac{\partial }{\partial x}({T}_{a}-{T}_{-h})\frac{\partial h}{\partial x}$$

A scaling of the three terms on the RHS of Eq. 6 suggests that the first term is higher than the other terms by an order of magnitude, and, therefore, we neglect the last two terms.

7 $${\int }_{-h}^{0}\,\frac{{\partial }^{2}T}{\partial {x}^{2}}dz\approx h\frac{{\partial }^{2}{T}_{a}}{\partial {x}^{2}}$$

Similarly,

8 $${\int }_{-h}^{0}\,\frac{{\partial }^{2}T}{\partial {y}^{2}}dz\approx h\frac{{\partial }^{2}{T}_{a}}{\partial {y}^{2}}$$

Substituting Eqs. 7 and 8 in 5, the horizontal mixing term averaged in the ML becomes,

9 $$\frac{{\kappa }_{H}}{h}\,{\int }_{-h}^{0}\,\left(\frac{{\partial }^{2}T}{\partial {x}^{2}}+\frac{{\partial }^{2}T}{\partial {y}^{2}}\right)dz={\kappa }_{H}\left(\frac{{\partial }^{2}{T}_{a}}{\partial {x}^{2}}+\frac{{\partial }^{2}{T}_{a}}{\partial {y}^{2}}\right)$$

The entrainment term has three components, as given below.

10 $$\left(\frac{{T}_{a}-{T}_{-h}}{h}\right)\left[\mathop{\underbrace{\frac{\partial h}{\partial t}}}\limits_{\text{ML}\,\text{tendency}}+\mathop{\underbrace{{u}_{-h}\frac{\partial h}{\partial x}+{v}_{-h}\frac{\partial h}{\partial y}}}\limits_{\text{Lateral}\,\text{induction}}+\mathop{\underbrace{{w}_{-h}}}\limits_{\text{Vertical}\,\text{advection}}\right]$$

The first term arises from the tendency (rate of change) of MLD. The second term results from horizontal advection across a sloping ML base and is often referred to as “lateral induction”^34^. The third term is the vertical advection.

Substituting Eqs. 4, 9 and 10 in Eq. 2 and re-arranging, we get,

11 $$\begin{array}{rcl}\mathop{\underbrace{\frac{\partial {T}_{a}}{\partial t}}}\limits_{\text{Tendency}} & = & \mathop{\underbrace{-\,\left({u}_{a}\frac{\partial {T}_{a}}{\partial x}+{v}_{a}\frac{\partial {T}_{a}}{\partial y}\right)}}\limits_{\text{Horizontal}\,\text{advection}}+\mathop{\underbrace{{\kappa }_{H}\left(\frac{{\partial }^{2}{T}_{a}}{\partial {x}^{2}}+\frac{{\partial }^{2}{T}_{a}}{\partial {y}^{2}}\right)}}\limits_{\text{Horizontal}\,\text{mixing}}-\mathop{\underbrace{\frac{1}{h}{\left[{\kappa }_{Z}\frac{\partial T}{\partial z}\right]}_{-h}}}\limits_{\text{Vertical}\,\text{mixing}}\\  &  & -\,\mathop{\underbrace{\left(\frac{{T}_{a}-{T}_{-h}}{h}\right)\left(\frac{\partial h}{\partial t}+{w}_{-h}+{u}_{-h}\frac{\partial h}{\partial x}+{v}_{-h}\frac{\partial h}{\partial y}\right)}}\limits_{\text{Entrainment}}+\mathop{\underbrace{\frac{{q}_{0}-{q}_{pen}}{{\rho }_{0}{c}_{p}h}}}\limits_{\text{Net}\,\text{heat}\,\text{flux}}\mathrm{}.\end{array}$$

### Estimation of terms

#### MLD

The MLD (h) was computed using a density criteria. It is defined as the depth at which the increase in density from its surface value corresponds to a decrease in temperature by 0.8 °C^21,35,36^. For a typical southern BoB salinity of 34 psu and temperature of 28 °C, a drop in temperature of 0.8 °C will result in an increase in potential density by 0.258 kg m^−3^. The analysis presented in George *et al*.^21^, with the same MLD criteria, using the VMP data collected during BoBBLE shows that the total kinetic energy (TKE) dissipation rate was greater than 10^−7^ W kg^−1^ in the ML, about two orders of magnitude higher than that was estimated just below the MLD. Further, they showed that a maxima in vertical shear estimated using the ADCP data coincided with the MLD. These results justify the MLD criteria chosen in this paper.

#### Tendency

The rate of change of temperature (tendency) at TSE was estimated from the CTD data. The data were first averaged in the ML and then the time derivative was estimated at hourly resolution using a centered differencing scheme. The evolution of SST was coherent with the ML temperature, but was marginally warmer by 0.06 °C on average, and exhibited stronger diurnal cycling (Fig. S2A). Note that we have deliberately chosen a definition of the ML that remains deeper than the diurnal warm layers, as these are not the focus of this study, so these differences are expected. The tendency of the SST (Fig. S2B) correlated well (0.90) with the tendency of the ML temperature (hereafter referred to as tendency).

#### Horizontal gradient from uCTD profiles

Any field such as temperature, T(x), varying in one dimension, can be expressed using the Taylor series expansion as follows,

12 $$T(x)=T({x}_{0})+{\frac{\partial T}{\partial x}|}_{{x}_{0}}\Delta x+{\frac{{\partial }^{2}T}{\partial {x}^{2}}|}_{{x}_{0}}\frac{\Delta {x}^{2}}{2}+\ldots $$

Applying a first order linear approximation, we can rewrite Eq. 12 as,

13 $$\Delta T=T(x)-T({x}_{0})={\frac{\partial T}{\partial x}|}_{{x}_{0}}\Delta x+O[\Delta {x}^{2}]$$

We made several estimates of Δ*T* utilizing temperature profiles measured by uCTD spread at different locations along a linear transect (see the schematic diagram in Fig. S3). Assuming that there is an offset of $\delta$ and a random variability of $\varepsilon (x)$, the observed temperature difference between the two points can be written as follows.

14 $$\Delta {T}_{obs}={T}_{uCT{D}_{i}}-{T}_{uCT{D}_{1}}=\frac{\partial T}{\partial x}\Delta x+\delta +\varepsilon (x)$$

where the offset $\delta$ is due to sampling variability and is typically very small (Figs. S4 and S5). The $\varepsilon (x)$ accounts for random variability and can be identified with the higher order terms O[Δ*x*^2^] from Eq. 13. Here, suffix i refers to the i^*th*^ uCTD profile along the linear transect and suffix 1 denotes the first profile, closest to TSE. A similar expression can be written for the meridional gradient of temperature in the y-direction. The observed Δ*T*_*obs*_ were then plotted as a function of Δ*x* using the westward uCTD section (Fig. S4) and Δ*y* using the southward section (Fig. S5) for each day. Straight lines were then fitted assuming that the scatter satisfies the first order approximation given in Eq. 13. The slope of the line (Eq. 14) gives the temperature gradient and the intercept gives the offset ($\delta$). Any scatter about the straight line will be due to random variability, including the higher order terms. This method of linear fitting assumes that the horizontal temperature gradient has a spatial scale of about 10 km and therefore any variability whose length scale is smaller than 10 km is an error. The temperature gradient along the meridional direction and the errors associated were also estimated in a similar way. We find that the data in Figs. S4 and S5 do lie approximately along straight lines, and conclude that the linear approximation made in Eq. 13 is valid. Here, we could get the estimates of the uncertainties involved in the gradient calculation, which is not possible with glider based gradient estimates. The offsets on 5 July in the estimate of $dT/dx$ and $dT/dy$ were −0.04 and −0.004 °C and the random variability were 0.03 and 0.01 °C. The offsets were removed while presenting the final estimate of the gradients.

#### Horizontal advection from glider profiles

We positioned the ship and two gliders in an L-shaped configuration (see Fig. S6). As the gliders are not stationary, their positions were different each time a profile was measured. Therefore, the temperature (averaged in ML) difference between a glider and CTD is given by

15 $$\Delta {T}_{1,2}={T}_{1,2}-{T}_{CTD}=\frac{\partial T}{\partial x}\Delta {x}_{1,2}+\frac{\partial T}{\partial y}\Delta {y}_{1,2}$$

Here, Δ${x}_{1,2}={x}_{1,2}-{x}_{CTD}$ and Δ${y}_{1,2}={y}_{1,2}-{y}_{CTD}$. Suffixes 1 and 2 denote the two different gliders. Solving for $\partial T/\partial x$ and $\partial T/\partial y$, we get

16 $$\frac{\partial T}{\partial x}=\frac{\Delta {T}_{2}\Delta {y}_{1}-\Delta {T}_{1}\Delta {y}_{2}}{\Delta {x}_{2}\Delta {y}_{1}-\Delta {x}_{1}\Delta {y}_{2}};\frac{\partial T}{\partial y}=\frac{\Delta {T}_{2}\Delta {x}_{1}-\Delta {T}_{1}\Delta {x}_{2}}{\Delta {y}_{2}\Delta {x}_{1}-\Delta {y}_{1}\Delta {x}_{2}}$$

If the observations were aligned on the x and y axes, Eq. 16 simplifies to the familiar finite difference estimates.

The averaged velocity components were then multiplied to the respective temperature gradients to obtain the components of horizontal temperature advection. Before averaging the ADCP-derived velocity components in the ML, it was assumed that the top 11 m (depth of the first bin of ADCP measurement) of the water column has a velocity equal to the velocity at 11 m. Alternative extrapolation methods were tested but found to be unrealistic.

#### Horizontal mixing

We utilize the uCTD data to estimate the second horizontal derivative of ML-averaged temperature. This method is similar to the method followed to compute the horizontal gradient from uCTD profiles, with difference in temperature replaced by a first derivative estimated using a forward difference scheme. We made several estimates of Δ*T*/Δ*x* utilizing temperature profiles measured by uCTD spread at different locations along the x-axis. Then Δ*T*/Δ*y* was plotted as a function of Δ*x* (similar to Eq. 14).

17 $${\frac{\varDelta T}{\varDelta x}}_{obs}=\frac{{T}_{uCT{D}_{i}}-{T}_{uCT{D}_{1}}}{\varDelta x}=\frac{\partial }{\partial x}\left(\frac{\partial T}{\partial x}\right)\varDelta x+\delta +\varepsilon (x)$$

The second derivative of temperature (${\partial }^{2}T/\partial {x}^{2}$) is the slope of the line that can be fitted to the observed scatter of Δ*T*/Δ*x* versus Δ*x*. *δ*, the intercept of the fit is the offset and $\varepsilon (x)$, measure of the scatter, is the random variability. The observed Δ*T*/Δ*x* and Δ*T*/Δ*y* were then plotted as functions of Δ*x* and Δ*y*, respectively (Figs. S7 and S8) leading to an estimate of ${\partial }^{2}T/\partial {x}^{2}$ of order 10^−9^ °C m^−2^.

${\kappa }_{H}$ is a function of length scale^37^. For a length scale of $10$ km, the order of magnitude of ${\kappa }_{H}$ is about 10 m^2^ s^−1^. The horizontal mixing term is then of order 10^−3^ °C day^−1^ and has a negligible contribution compared to horizontal advection (Fig. 4F). Earlier studies also suggest that this term is very small^38–40^.

#### Vertical mixing

To estimate the vertical diffusion coefficient using the VMP data, we followed the methodology described in George *et al*.^21^. Turbulent kinetic energy dissipation rate ($\varepsilon$) was estimated from the velocity shears measured by the VMP following Roget *et al*.^41^ assuming isotopic turbulence. The vertical diffusion coefficient was then calculated using the relationship^42^, *K*_*Z*_ = Γ$\varepsilon /{N}^{2}$. The mixing efficiency (Γ) was taken to be a constant (0.2) and the Brunt-Väisälä frequency (${N}^{2}$) was estimated using the CTD data. The finite difference decomposition of the vertical mixing term (Eq. 4) was carried out using a forward difference scheme applied at the base of the ML.

#### Entrainment

The tendencies of the MLD and the depth of 27 °C isotherm were obtained by applying a forward difference scheme. The gradient of MLD was estimated using an equation similar to Eq. 16, the variable temperature (T) being replaced with MLD (h).

Vertical advection was estimated as the tendency of 27 °C isotherm located in the thermocline, assuming that the base of the ML oscillates at the same rate as the thermocline^43^. The assumption here is that entrainment occurs when there is a difference in the vertical movement of the ML and the vertical movement of the thermocline. By adding the ML tendency and the vertical advection term (which have opposite signs), we compute the difference in vertical motion of these two surfaces. We assume that this difference in vertical motion reflects the entrainment of water into the ML, since adiabatic waves will move both surfaces in concert. Internal waves that stretch or compress both the thermocline and the ML do not lead to a transport of heat through the base of the ML and thus do not directly influence ML temperature. In short, the assumption is that the ocean is adiabatic in the interior and the water parcels do not cross the pycnocline.

#### Net surface heat flux

Estimation of sensible and turbulent latent heat fluxes were done using the eddy covariance method^44–46^.

#### Penetrating short wave radiation

*q*_*pen*_ was estimated based on Lotliker *et al*.^47^. The diffuse attenuation coefficient of photosynthetically available radiation (PAR) at wavelengths between 400–700 nm (${k}_{PAR}$) was estimated using the following equation.

18 $$PAR(z)={\int }_{400}^{700}\,{E}_{d}(z,\lambda )d\lambda =({\int }_{400}^{700}\,{E}_{d}({0}^{+},\lambda )d\lambda ){e}^{{k}_{PAR}z}$$

Here, ${E}_{d}(z,\lambda )$ is the spectral downwelling irradiance at depth $z$ and wavelength $\lambda$ measured using the hyperspectral profiling radiometer. ${E}_{d}({0}^{+},\lambda )$ represents the spectral downwelling irradiance just above the sea surface and was calculated using the Fresnel reflection albedo ($\alpha =0.043$) for irradiance from sun and sky as given below.

19 $${E}_{d}({0}^{+},\lambda )=\frac{{E}_{d}({0}^{-},\lambda )}{1-\alpha }.$$

${E}_{d}({0}^{-},\lambda )$ represents the spectral downwelling irradiance just below the sea surface. ${k}_{PAR}$ varied between 0.0635 to 0.0759 m^−1^ during the time-series observation at TSE. The daily values of ${k}_{PAR}$ were then used to fit exponential curves and were compared with downwelling irradiance averaged over the visible spectrum during 4–13 July 2016 at TSE (see Fig. S9). Finally, the penetrative short wave loss at the base of the ML was estimated as a function of the net shortwave radiation received at the sea surface.

20 $${q}_{pen}={q}_{sw}{e}^{-{k}_{PAR}h}$$

It was found that the estimated *q*_*pen*_ inversely correlated (−0.81 with 99% significance) with the variation of chlorophyll in the ML (Fig. S1).

## Supplementary information

Supplementary Information.

## References

[1] Gadgil S, Joseph PV, Joshi NV (1984). Ocean–atmosphere coupling over monsoon regions. Nature.

[2] Graham NE, Barnett TP (1987). Sea surface temperature, surface wind divergence, and convection over tropical oceans. Science.

[3] Gadgil S (2003). The Indian monsoon and its variability. Annu. Rev. Earth Planet. Sci..

[4] Cane MA (1983). Oceanographic events during El Niño. Science.

[5] Knutson TR (2010). Tropical cyclones and climate change. Nat. Geosci..

[6] Sriver RL, Huber M (2007). Observational evidence for an ocean heat pump induced by tropical cyclones. Nature.

[7] Meehl GA, Arblaster JM, Bitz CM, Chung CTY, Teng H (2016). Antarctic sea-ice expansion between 2000 and 2014 driven by tropical Pacific decadal climate variability. Nat. Geosci..

[8] Cai, W. *et al*. Pantropical climate interactions. *Science* **363**, 10.1126/science.aav4236 (2019). 10.1126/science.aav4236 30819937

[9] Wang, W. & McPhaden, M. J. The surface-layer heat balance in the equatorial Pacific Ocean. Part I: Mean seasonal cycle. *J*. *Phys*. *Oceanogr*. **29**, 1812–1831, 10.1175/1520-0485(1999)029<1812:TSLHBI>2.0.CO;2 (1999).

[10] Foltz, G. R., Grodsky, S. A., Carton, J. A. & McPhaden, M. J. Seasonal mixed layer heat budget of the tropical Atlantic Ocean. *J*. *Geophys*. *Res*. *Ocean*. **108**, 10.1029/2002JC001584, 3146 (2003).

[11] McPhaden MJ, Foltz GR (2013). Intraseasonal variations in the surface layer heat balance of the central equatorial Indian Ocean: The importance of zonal advection and vertical mixing. Geophys. Res. Lett..

[12] Wijesekera, H. W. *et al*. Upper ocean heat and freshwater budgets in the eastern Pacific warm pool. *J*. *Geophys*. *Res*. *Ocean*. **110**, 10.1029/2004JC002511 (2005).

[13] McPhaden MJ (2009). RAMA: The Research Moored Array for African-Asian-Australian monsoon analysis and prediction. Bull. Am. Meteorol. Soc..

[14] McPhaden MJ (1998). The Tropical Ocean-Global Atmosphere observing system: A decade of progress. J. Geophys. Res. Ocean..

[15] Bourlès B (2008). The PIRATA program: History, accomplishments, and future directions. Bull. Am. Meteorol. Soc..

[16] Morel, A. & Antoine, D. Heating rate within the upper ocean in relation to its bio–optical state. *J*. *Phys*. *Oceanogr*. **24**, 1652–1665, 10.1175/1520-0485(1994)024<1652:HRWTUO>2.0.CO;2 (1994).

[17] Feng M, Hacker P, Lukas R (1998). Upper ocean heat and salt balances in response to a westerly wind burst in the western equatorial Pacific during TOGA COARE. J. Geophys. Res. Ocean..

[18] Vinayachandran PN (2018). BoBBLE (Bay of Bengal Boundary Layer Experiment): Ocean-atmosphere interaction and its impact on the south Asian monsoon. Bull. Am. Meteorol. Soc..

[19] Vinayachandran, P. N. *et al*. Maintenance of the southern Bay of Bengal cold pool. *Deep*. *Sea Res*. *Part II*: *Top*. *Stud*. *Oceanogr*. 104624, 10.1016/j.dsr2.2019.07.012 (2019).

[20] Webber BGM (2018). The dynamics of the southwest monsoon current in 2016 from high-resolution *in situ* observations and models. J. Phys. Oceanogr..

[21] George JV (2019). Vertical mixing during barrier layer formation and erosion: Observation from Bay of Bengal. J. Phys. Oceanogr..

[22] Matthews AJ, Baranowski DB, Heywood KJ, Flatau PJ, Schmidtko S (2014). The surface diurnal warm layer in the Indian Ocean during CINDY/DYNAMO. J. Clim..

[23] Vialard, J. *et al*. Processes of 30–90 days sea surface temperature variability in the northern Indian Ocean during boreal summer. *Clim*. *Dyn*. **38**, 10.1007/s00382-011-1015-3 (2012).

[24] Joseph PV, Sooraj KP, Babu CA, Sabin TP (2005). A cold pool in the Bay of Bengal and its interaction with the active-break cycle of the monsoon. CLIVAR Exch..

[25] Shankar D, Shetye SR, Joseph PV (2007). Link between convection and meridional gradient of sea surface temperature in the Bay of Bengal. J. Earth Syst. Sci..

[26] Goswami BN (1987). A mechanism for the west-northwest movement of monsoon depressions. Nature.

[27] Levine RC, Turner AG (2012). Dependence of Indian monsoon rainfall on moisture fluxes across the Arabian Sea and the impact of coupled model sea surface temperature biases. Clim. Dyn..

[28] Li Y (2017). Bay of Bengal salinity stratification and Indian summer monsoon intraseasonal oscillation: 2. Impact on SST and convection. J. Geophys. Res. Ocean..

[29] Zhang, C. Madden-Julian Oscillation. *Rev*. *Geophys*. **43**, 10.1029/2004RG000158 (2005).

[30] Rasmusson, E. M. & Carpenter, T. H. Variations in tropical sea surface temperature and surface wind fields associated with the Southern Oscillation/El Niño. *Mon*. *Weather*. *Rev*. **110**, 354–384, 10.1175/1520-0493(1982)110<0354:VITSST>2.0.CO;2 (1982).

[31] Saji NH, Goswami BN, Vinayachandran PN, Yamagata T (1999). A dipole mode in the tropical Indian Ocean. Nature.

[32] Firing, E. & Hummon, J. M. Shipboard ADCP measurements. In Hood, E., Sabine, C. & Sloyan, B. (eds) *Shipboard ADCP Measurements*, *In*: *The GO*-*SHIP Repeat Hydrography Manual*: *A Collection of Expert Reports and Guidelines*. *Version 1*, 11pp (ICPO Publication Series, IOCCP Report Number 14, 2010).

[33] Moisan, J. R. & Niiler, P. P. The seasonal heat budget of the north Pacific: Net heat flux and heat storage rates (1950–1990). *J*. *Phys*. *Oceanogr*. **28**, 401–421, 10.1175/1520-0485(1998)028<0401:TSHBOT>2.0.CO;2 (1998).

[34] Kim S-B, Fukumori I, Lee T (2006). The closure of the ocean mixed layer temperature budget using level-coordinate model fields. J. Atmospheric Ocean. Technol..

[35] Girishkumar, M. S., Ravichandran, M., McPhaden, M. J. & Rao, R. R. Intraseasonal variability in barrier layer thickness in the south central Bay of Bengal. *J*. *Geophys*. *Res*. *Ocean*. **116**, 10.1029/2010JC006657 (2011).

[36] Kara AB, Rochford PA, Hurlburt HE (2000). An optimal definition for ocean mixed layer depth. J. Geophys. Res. Ocean..

[37] Okubo A (1971). Oceanic diffusion diagrams. Deep. Sea Res. Oceanogr. Abstr..

[38] Kurian, J. & Vinayachandran, P. N. Mechanisms of formation of the Arabian Sea mini warm pool in a high-resolution ocean general circulation model. *J*. *Geophys*. *Res*. *Ocean*. **112**, 10.1029/2006JC003631, C05009 (2007).

[39] Gill AE, Niiler PP (1973). The theory of the seasonal variability in the ocean. Deep. Sea Res. Oceanogr. Abstr..

[40] Hansen DV, Paul CA (1984). Genesis and effects of long waves in the equatorial Pacific. J. Geophys. Res. Ocean..

[41] Roget E, Lozovatsky I, Sanchez X, Figueroa M (2006). Microstructure measurements in natural waters: Methodology and applications. Prog. Oceanogr..

[42] Osborn, T. R. Estimates of the local rate of vertical diffusion from dissipation measurements. *J*. *Phys*. *Oceanogr*. **10**, 83–89, 10.1175/1520-0485(1980)010<0083:EOTLRO>2.0.CO;2 (1980).

[43] McPhaden MJ (1982). Variability in the central equatorial Indian Ocean, part ii: Oceanic heat and turbulent energy balance. J. Mar. Res..

[44] Fairall, C. W., White, A. B., Edson, J. B. & Hare, J. E. Integrated shipboard measurements of the marine boundary layer. *J*. *Atmospheric Ocean*. *Technol*. **14**, 338–359, 10.1175/1520-0426(1997)014<0338:ISMOTM>2.0.CO;2 (1997).

[45] Edson, J. B., Hinton, A. A., Prada, K. E., Hare, J. E. & Fairall, C.W. Direct covariance flux estimates from mobile platforms at sea. *J*. *Atmospheric Ocean*. *Technol*. **15**, 547–562, 10.1175/1520-0426(1998)015<0547:DCFEFM>2.0.CO;2 (1998).

[46] Dupuis, H. *et al*. Impact of flow distortion corrections on turbulent fluxes estimated by the inertial dissipation method during the FETCH experiment on R/V L’Atalante. *J*. *Geophys*. *Res*. *Ocean*. **108**, 10.1029/2001JC001075, 8064 (2003).

[47] Lotliker, A. A. *et al*. Penetrative radiative flux in the Bay of Bengal. *Oceanography* **29**, 10.5670/oceanog.2016.53 (2016).

