# Supplementary material for: Closing the sea surface mixed layer temperature budget from in situ observations alone: Operation Advection during BoBBLE
Source: Sci Rep. 2020 Apr 27;10:7062. doi: 10.1038/s41598-020-63320-0 (PMC7184617; doi:10.1038/s41598-020-63320-0)
Supplement: Supplementary file 1 — Supplementary Information. [file 41598_2020_63320_MOESM1_ESM.pdf]

Closing the sea surface mixed layer temperature budget from in-situ observations alone: Operation Advection during BoBBLE

V. Vijith[1,2], P. N. Vinayachandran[1,*], Benjamin G. M. Webber[3], Adrian J. Matthews[4], Jenson V. George[1], Vijay Kumar Kannaujia[5], Aneesh A. Lotliker[6], and P. Amol[7]

[1]Centre for Atmospheric and Oceanic Sciences, Indian Institute of Science, Bangalore, India
[2]School of Marine Sciences, Cochin University of Science and Technology, Kochi, India (present affiliation)
[3]Climatic Research Unit, School of Environmental Sciences, Centre for Ocean and Atmospheric Sciences, University of East Anglia, UK
[4]School of Environmental Sciences and School of Mathematics, Centre for Ocean and Atmospheric Sciences, University of East Anglia, UK
[5]CSIR-National Institute of Oceanography, Goa, India
[6]Indian National Centre for Ocean Information Services, Hyderabad, India
[7]CSIR-National Institute of Oceanography, Regional Center, Visakhapatnam, India
[*]vinay@iisc.ac.in

ABSTRACT

This document includes supplementary figures.

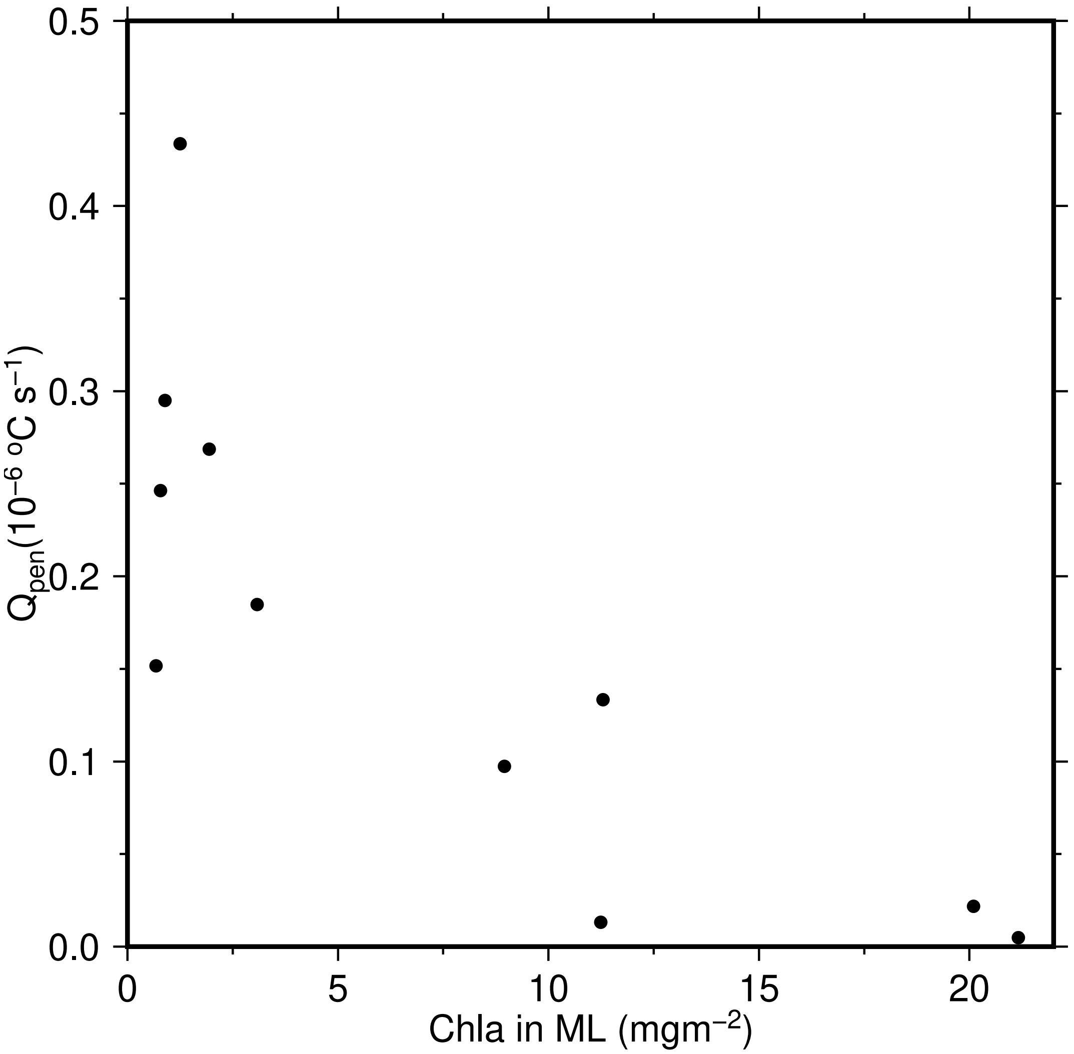

Figure S1. Daily averaged penetrative short-wave radiation at the base of the ML as a function of total chlorophyll in the ML.

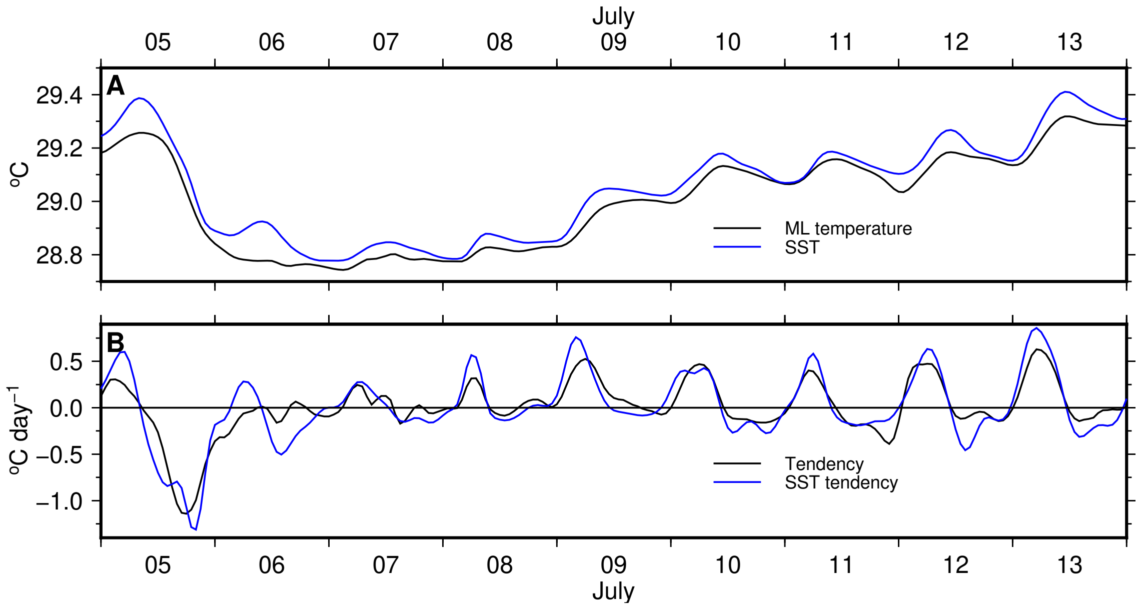

Figure S2. Time series of ML-averaged temperature and SST, (B) tendencies of the ML-averaged temperature and SST. All the variables are smoothed using a 4-hour moving window to remove high-frequency noise.

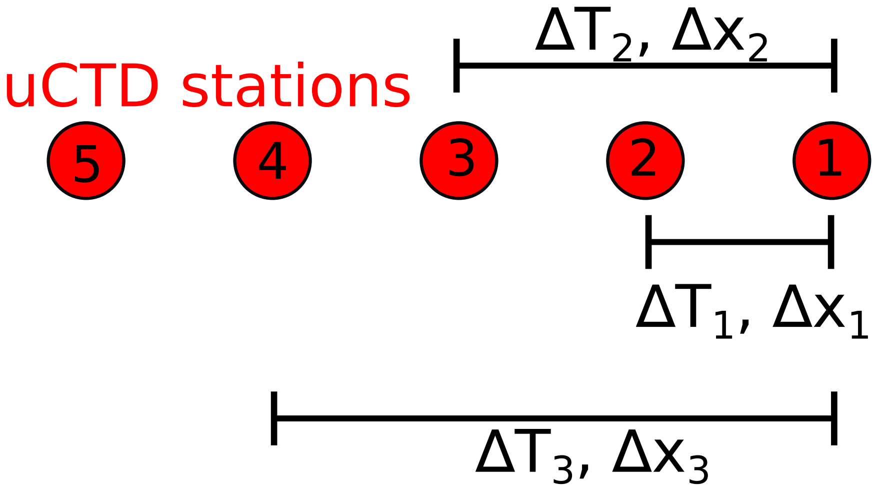

Figure S3. Schematic diagram illustrating the gradient calculation using the uCTD section. ΔT_i denote the difference of mixed-layer averaged temperature at each uCTD station with the temperature at the first uCTD station. Δx_i denote the distance between each uCTD station from the first station. Suffix, i=1,5, denote the uCTD station number.

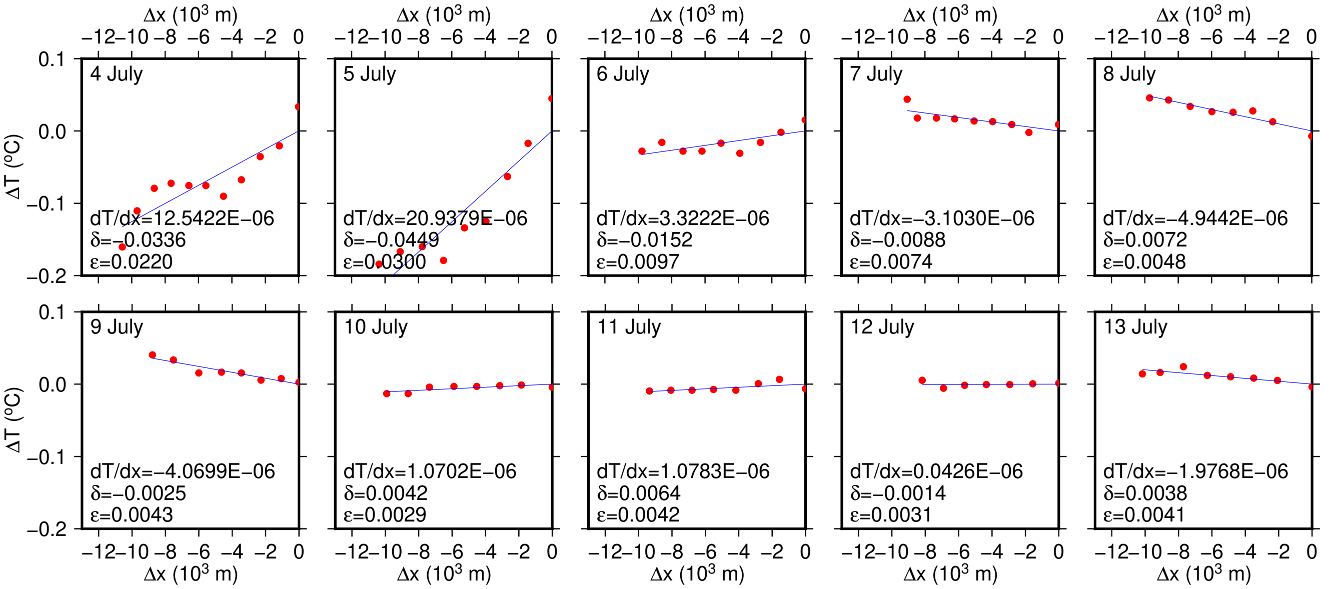

Figure S4. ΔT plotted as a function of Δx for each day of measurement using the westward leg of the uCTD. The method of computation of the differences is shown in Fig. S3. The slope of the straight-line fit is the gradient in the zonal direction ($\frac{\partial T}{\partial x}$), δ is the offset and ε is the root mean square value of the random variability. Note that δ is removed from each of these fits.

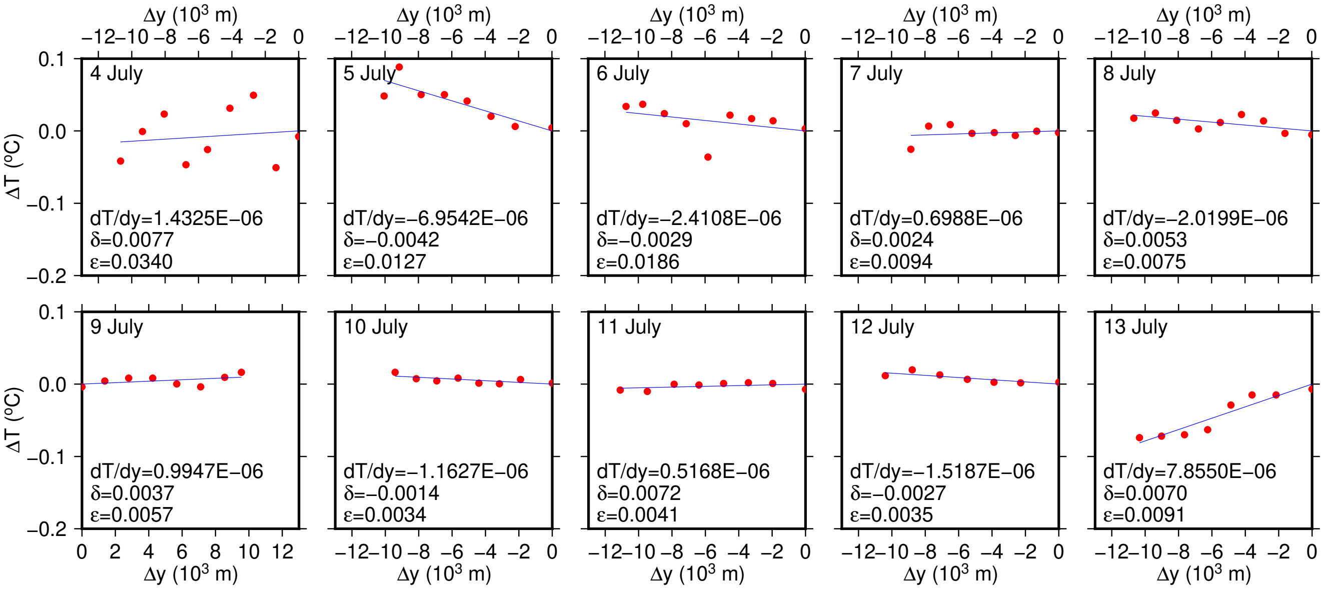

Figure S5. Same as Fig. S4. But using the southward leg of the uCTD for estimating the meridional gradient of temperature ($\frac{\partial T}{\partial y}$).

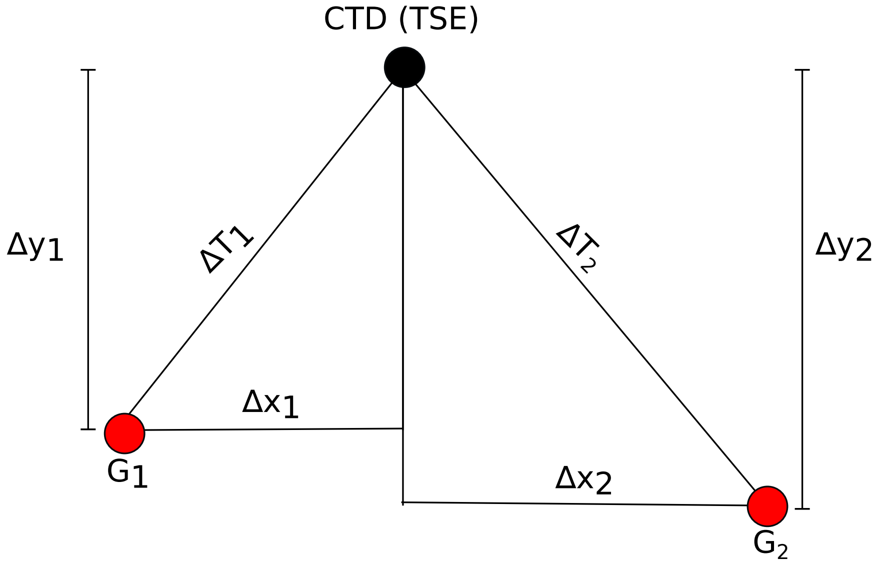

Figure S6. Schematic diagram illustrating the gradient calculation based on the ship-glider triangular configuration. ΔT_i denote the difference of mixed-layer averaged temperature at each glider station with the temperature at TSE measured using CTD. Δx_i ad Δy_i denote the zonal and meridional distance, respectively, between each glider from TSE.

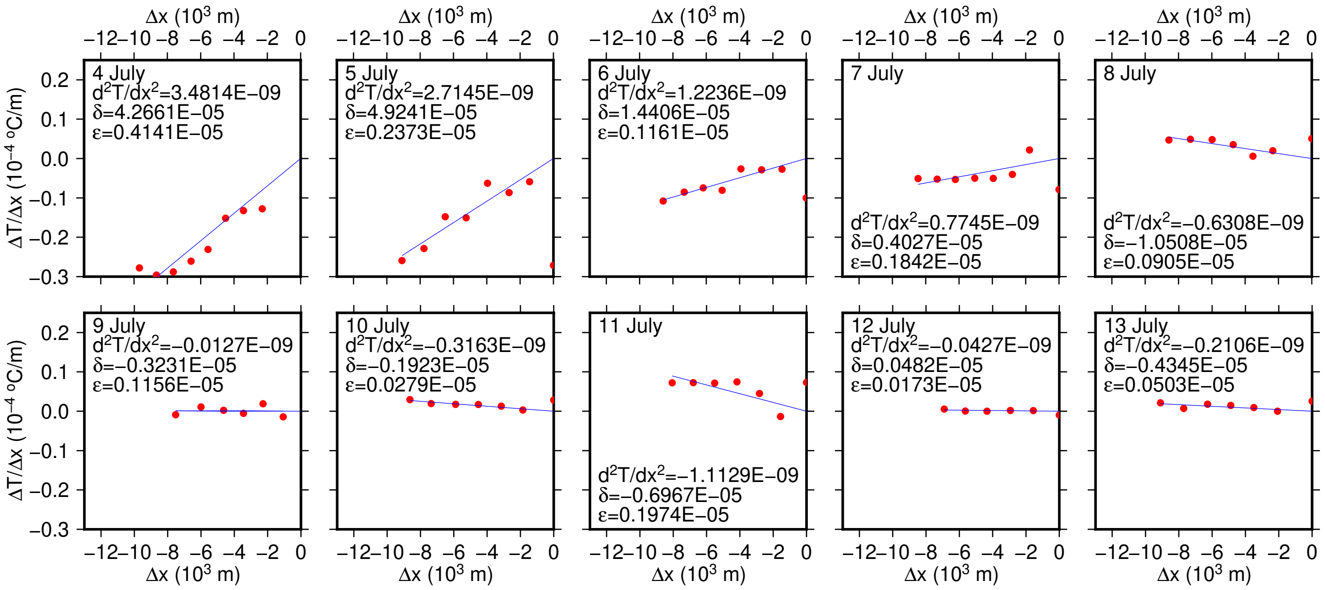

Figure S7. $\Delta T/\Delta x$ differences plotted as a function of Δx for each day of measurement using the westward leg of the uCTD. The slope of the straight-line fit is the second derivative of temperature in the zonal direction ($\frac{\partial^2 T}{\partial x^2}$), δ is the offset and ε is the root mean square of the random variability. Note that δ is removed from each of these fits.

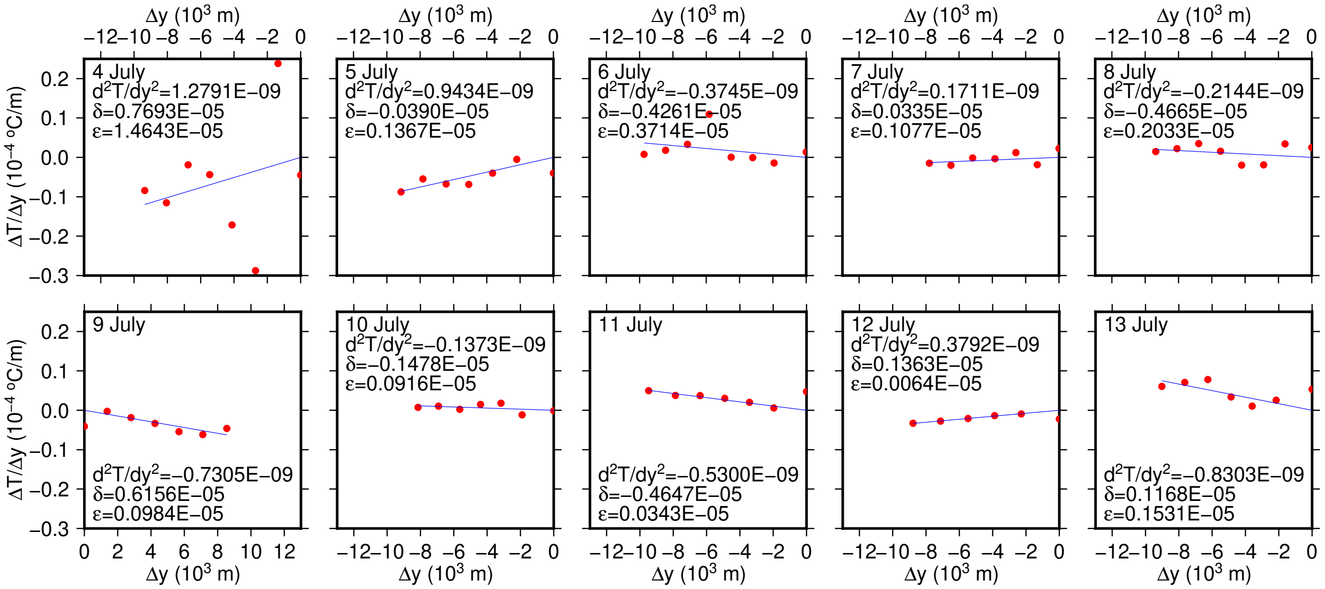

Figure S8. Same as Fig. S7. But using the southward leg of the uCTD for estimating the second derivative of temperature in the zonal direction ($\frac{\partial^2 T}{\partial y^2}$).

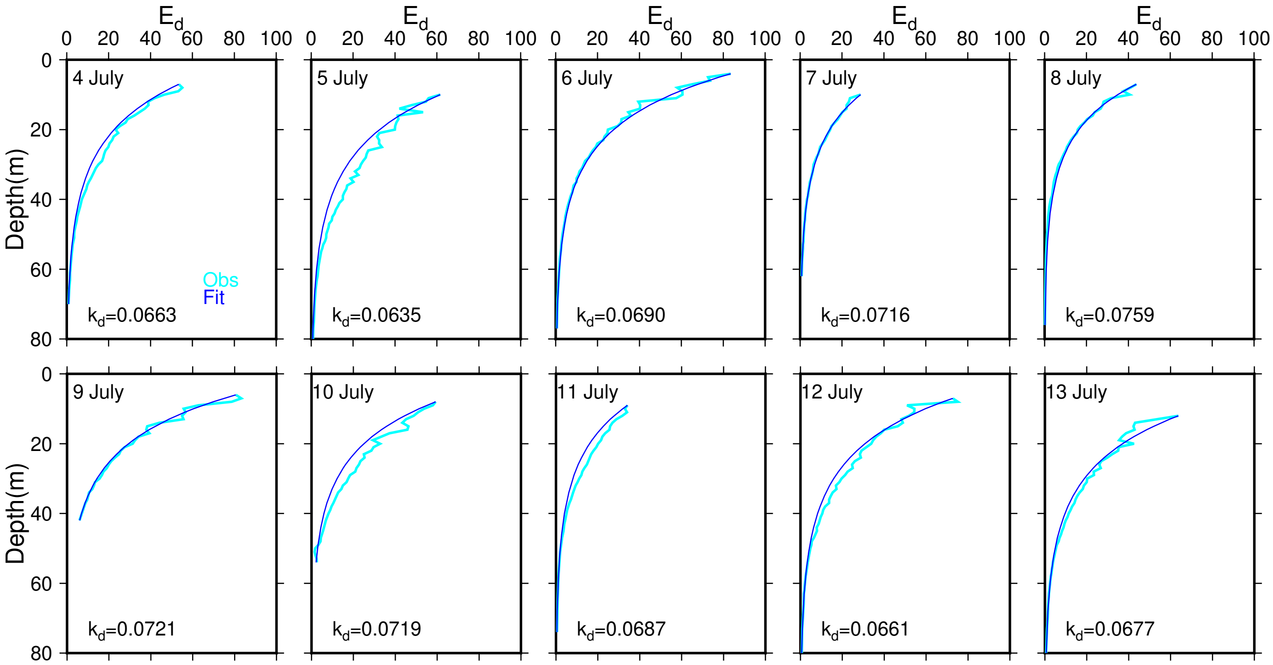

Figure S9. Comparison of downwelling irradiance averaged over the visible spectrum (400–700 nm) measured during the time-series observation at TSE with an exponential fit based on an averaged k_{PAR} equal to 0.0682.
